# The Hidden Link between Polycystic Ovary Syndrome and Kidney Stones: Finding from the Tehran Lipid and Glucose Study (TLGS)

**DOI:** 10.3390/diagnostics13172814

**Published:** 2023-08-30

**Authors:** Marzieh Rostami Dovom, Maryam Rahmati, Saber Amanollahi Soudmand, Pardis Ziaeefar, Fereidoun Azizi, Fahimeh Ramezani Tehrani

**Affiliations:** 1 Reproductive Endocrinology Research Center, Research Institute for Endocrine Sciences, Shahid Beheshti University of Medical Sciences, Tehran 1985717413, Iran; marzieh.rostamidovom@gmail.com (M.R.D.); rahmatimary@gmail.com (M.R.); 2 Urology Department, Labafinejad Hospital, School of Medicine, Shahid Beheshti University of Medical Sciences, Tehran 1985717443, Iran; saberas_2008@yahoo.com; 3 School of Medicine, Shahid Beheshti University of Medical Sciences, Tehran 1985717443, Iran; pards.ziaeefar@gmail.com; 4 Endocrine Research Center, Research Institute for Endocrine Sciences, Shahid Beheshti University of Medical Sciences, Tehran 1985717413, Iran; azizi@endocrine.ac.ir

**Keywords:** polycystic ovarian syndrome (PCOS), kidney stone disease, nephrolithiasis, menstrual irregularities, hyperandrogenism, Tehran lipid and glucose study (TLGS)

## Abstract

Background: We aimed to investigate the association between kidney stones and polycystic ovarian syndrome (PCOS). Materials and methods: In a cross-sectional study, data from the Tehran Lipid and Glucose Study (TLGS) were used to investigate the risk of kidney stones in women with Polycystic Ovary Syndrome (PCOS). Four distinct phenotypes of PCOS, as defined by the Rotterdam criteria, were examined in a sample of 520 women and compared to a control group of 1638 eumenorrheic non-hirsute healthy women. Univariate and multivariable logistic regression models were employed for analysis. The four PCOS phenotypes were classified as follows: Phenotype A, characterized by the presence of all three PCOS features (anovulation (OA), hyperandrogenism (HA), and polycystic ovarian morphology on ultrasound (PCOM)); Phenotype B, characterized by the presence of anovulation and hyperandrogenism; Phenotype C, characterized by the presence of hyperandrogenism and polycystic ovarian morphology on ultrasound; and Phenotype D, characterized by the presence of anovulation and polycystic ovarian morphology on ultrasound. Results: The prevalence of a history of kidney stones was found to be significantly higher in women with Polycystic Ovary Syndrome (PCOS) compared to healthy controls (12.5% vs. 7.7%, *p* = 0.001). This increased prevalence was observed across all PCOS phenotypes (*p* < 0.001). After adjusting for potential risk factors, including age, family history of kidney stones, waist-to-height ratio, total cholesterol, and low-density lipoprotein, the odds ratio for kidney stones in women with PCOS was found to be 1.59 [95% CI: 1.12–2.25, *p* = 0.01], indicating a 59% increase in risk compared to healthy women. Women with PCOS Phenotype A [OR: 1.97, 95% CI: 1.09–3.55, *p* = 0.02] and Phenotype D [OR: 3.03, 95% CI: 1.24–7.41, *p* = 0.01] were found to be at a higher risk for kidney stones. Conclusion: Women with Polycystic Ovary Syndrome (PCOS), particularly those exhibiting menstrual irregularities and polycystic ovarian morphology on ultrasound (PCOM), have been found to be two to three times more likely to develop kidney stones. This increased prevalence should be taken into consideration when providing preventive care and counseling to these individuals.

## 1. Introduction

Polycystic Ovary Syndrome (PCOS) is a prevalent endocrine disorder affecting women of reproductive age, characterized by a combination of clinical manifestations including hyperandrogenism and/or hyperandrogenemia, oligo/anovulation, and polycystic ovarian morphology as observed on ultrasonography. The worldwide prevalence of PCOS is estimated to be between 6 and 12% [1,2]. This disorder is primarily caused by the overproduction of androgens by the ovaries and, in some cases, the adrenal glands, leading to a variety of clinical features including menstrual disorders and increased symptoms of male hormones such as hirsutism, acne, oily skin, and androgenic alopecia [3,4,5,6,7]. Hyperandrogenism and/or hyperandrogenemia are the most common diagnostic features of PCOS [5], present in more than 82% of patients [8,9]. Many PCOS patients are obese, and lifestyle changes such as weight loss and abdominal fat reduction have been shown to be associated with decreased testosterone levels and increased insulin sensitivity, leading to improvement in hirsutism [10].

The relationship between kidney stones and polycystic ovaries has not been directly addressed in any study, but this hypothesis has been proposed [11,12]. Kidney Stone Disease (KSD) is a prevalent condition affecting the urinary tract, with an estimated incidence of 1–5% among Asians, 5–9% among Europeans, and 13–17% among North Americans [13]. The incidence of KSD is two to three times higher in men compared to women, suggesting a potential role of male androgen hormones, specifically testosterone, in the formation of calcium oxalate kidney stones [14,15,16,17]. Low testosterone levels have been identified as a protective factor against the formation of oxalate stones in the urinary tract in women and children [18]. Animal studies in rats have further supported this association, demonstrating that testosterone consumption increases the excretion of urinary oxalate and promotes the formation of calcium oxalate stones [19]. Testosterone replacement therapy has also been shown to increase the absolute risk of urinary stone formation when compared to individuals with those not undergoing hormone therapy [20].

This study aimed to investigate the relationship between Kidney Stone Disease (KSD) and Polycystic Ovary Syndrome (PCOS), as well as different PCOS phenotypes.

## 2. Material and Methods

### 2.1. Study Population and Variables

This study was approved by the ethical review board of the Research Institute for Endocrine Sciences (Code: IR.SMBU.ENDOCRINE.REC.1398) and informed consent was obtained from all participants. Data from the Tehran Lipid and Glucose Study (TLGS) were utilized for the purposes of this study. The TLGS, initiated in 1998, aimed to assess the risk factors of non-communicable diseases in an urban population. A total of 15,005 individuals aged ≥3 years participated in the TLGS, and data on demographic status; anthropometric, reproductive, hormonal, and cardio-metabolic characteristics; as well as general physical examinations and laboratory measurements were collected. Details of TLGS procedures have been reported previously [21]. For the present study, all women aged 18–45 years with available data on PCOS and kidney stone status were included. Information on demographic variables, obstetrics, and reproductive history was collected through a standard comprehensive questionnaire during face-to-face interviews. General anthropometrics and physical examinations included an assessment of hirsutism using the modified Ferriman–Gallwey scoring method by a trained general physician. The history of kidney stones and family history of kidney stones were assessed through face-to-face interviews or by observing related documents. Transvaginal or trans-abdominal ovarian ultrasound was performed using a 3.5 MHz trans-abdominal and 5 MHz transvaginal transducer by an experienced sonographer on the same day as the blood sample collection. Blood samples were taken between 7:00 and 9:00 a.m. after 12 h of overnight fasting during the early follicular phase of a spontaneous or progesterone-induced menstrual cycle. All sera were stored at −80 °C until the time of measurements.

### 2.2. Terms’ Definitions

PCOS was defined according to the Rotterdam criteria as the presence of two or more of the following criteria: oligo/anovulation, clinical or biochemical hyperandrogenism, and polycystic ovarian morphology. Oligo/anovulation was defined as either regular or irregular menstrual cycles >35 days or those with a history of eight or fewer menstrual cycles in a year. Clinical hyperandrogenism was defined as hirsutism, acne, or androgenic alopecia. Hirsutism was assessed based on the Freeman–Gallwey scoring system by observing hair growth in nine areas on the body that are typically without hair or with fluffy hair in most women. These areas included the upper lip, chin, chest, upper umbilicus, lower umbilicus, arms, thighs, back of the chest, back, and buttocks. A score of zero (minimum hair) to four (maximum hair) was assigned for each area [22,23]. In this study, individuals with a score ≥8 were considered to have hirsutism. Oligo/anovulation was considered as an increased interval between menstruations greater than 35 days or eight or fewer menstrual periods per year. PCOM was defined as observing ≥12 follicles measuring 2–9 mm in diameter and/or an ovarian volume >10 mL in at least one ovary. Four different phenotypes of PCOS were considered: Phenotype A—individuals with all three features of PCOS (oligo/anovulation + hyperandrogenism + polycystic ovarian morphology); Phenotype B—individuals with two features of PCOS (oligo/anovulation + hyperandrogenism); Phenotype C—individuals with two features of PCOS (hyperandrogenism + polycystic ovarian morphology); Phenotype D—individuals with two features of PCOS (oligo/anovulation + polycystic ovarian morphology).

History of kidney stones was assessed by asking them “Has any physician ever told you that you have kidney stones? If yes do you have confirmatory documents?” Family history of the kidney stones was assessed by asking them “Have your first-degree relatives ever been diagnosed with kidney stones?”

### 2.3. Hormonal Assessments

Luteinizing hormone (LH) and follicle-stimulating hormone [24] were measured through immunoradiometric assay [23] (Izotop, Budapest, Hungary) using gamma counter (Wallac Wizard, Turku, Finland). 17-hydroxyprogesterone (17OH-P), total testosterone (TT), androstenedione (A4), and dehydroepiandrosterone sulfate (DHEAS) were measured using enzyme immunoassay (EIA) (Diagnostic Biochem Canada Co., Ltd., London, ON, Canada). Sex Hormone Binding Globulin (SHBG) was measured using immunoenzymometric assay (IEMA) (Mercodia, Uppsala, Sweden). Free androgen index (FAI) was calculated using the formula (TT (nmol/L) × 100/SHBG (nmol/L)). All ELISA tests were performed using Sunrise ELISA reader (Tecan Co. Salzburg, Austria). The intra- and inter-assay coefficients of variation (CVs) for LH were 3% and 5.8%; for FSH: 3.5% and 4%; for 17 OH-P: 4.8% and 6.8%; for TT: 5.6% and 6.6%; for A4: 2.2% and 3.5%; for DHEAS: 2.0% and 5.1%; and for SHBG: 1.2% and 5.7%, respectively.

### 2.4. Statistical Analysis

Continuous variables were checked for normality based on the one-sample Kolmogorov–Smirnov test and are presented as mean (standard deviation) if they had a normal distribution, or median with inter-quartile range (Q25–Q75) for variables with skewed distribution. Categorical variables are presented as numbers and percentages. Characteristics of women were compared between the groups, by applying the two-independent-sample *t*-test (or ANOVA) and Pearson’s chi-square test for continuous and categorical data, respectively. The Mann–Whitney U test (or Kruskal–Wallis test) was applied to compare variables with skewed distribution. The Bonferroni post hoc test or Dunn test was used for significant ANOVA and Kruskal–Wallis variables, respectively.

In order to explore the effect of PCOS status and its phenotypes on the history of kidney stones, univariate and multivariable logistic regression models were applied, and odds ratios with 95% confidence intervals were estimated. Each independent variable was examined by a univariate model and those associated with the study outcome (*p* < 0.1) would be included in the multivariable model. 

According to the final multivariable logistic regression model and by using the nomolog function in STATA version 17, the nomogram model was constructed. The values for model covariates were mapped to points in the range of 0 to 10. The total number of scores obtained by the predictive model corresponded to the probability of kidney stone disease. To determine the predicted probability of kidney stones using a nomogram, one must first locate the patient’s values on each axis for each variable. By drawing a vertical line from these values to the “Score” axis, one can determine the corresponding score attributed to each variable value. The scores for all variables are then totaled. The sum is then located on the “Total score” line, and another vertical line is drawn to calculate the probability of kidney stones for the specific patient on the “Probability of kidney stones” axis. Two approaches were employed to assess the external validation of the proposed nomogram. Firstly, discrimination was evaluated using the area under the receiver operating characteristic curve (AUC) and the C index. A C index of 0.5 indicated a lack of predictive effect, while a C index of 1 indicated complete concordance between predicted and actual results. A higher C index value indicated better predictive accuracy [25]. Secondly, the calibration was assessed using the Hosmer–Lemeshow goodness-of-fit test. A smaller chi-square value and a larger corresponding *p*-value indicated better calibration. A statistically significant result (*p* < 0.05) suggested discrepancies between predicted and observed values, indicating poor model calibration [26]. Bias-corrected estimates of predictive accuracy were obtained through 200 bootstraps resampled to internally validate the nomograms. The performance characteristic of the nomograms was graphically analyzed using calibration plots, with the x-axis representing the predicted probability and the y-axis representing the observed proportion of kidney stones. Statistical analysis was conducted using STATA software (version 17.3; STATA Inc., College Station, TX, USA) and a significance level of *p* < 0.05 was used.

## 3. Results

This study included a total of 520 women diagnosed with Polycystic Ovary Syndrome (PCOS) and 1638 healthy women as controls. The prevalence of each PCOS phenotype among the sample was as follows: Phenotype A—23.9% (124/520), Phenotype B—48.1% (250/520), Phenotype C—21.0% (108/520), and Phenotype D—7.31% (38/520).

Table 1 presents the characteristics of women with PCOS and healthy controls. Compared to healthy women, those with PCOS were found to be younger (26.0 vs. 28.4 years, *p* < 0.001) and had a lower waist-to-height ratio (WHtR) and A body shape index (ABSI) (WHtR: 0.50 vs. 0.51, *p* = 0.02 and ABSI: 0.73 vs. 0.74, *p* = 0.001, respectively). The prevalence of a history of kidney stones was significantly higher in PCOS women (12.5%) compared to healthy controls (7.7%, *p* = 0.001).

Table 2 presents the comparison of demographic and other characteristics of women with different PCOS phenotypes (A, B, C, and D) versus healthy controls. The prevalence of kidney stone history among women with all PCOS phenotypes, except B, was significantly higher than in healthy women (*p* = 0.01). Additionally, the prevalence of a family history of kidney stones in all four PCOS phenotypes was also found to be significantly higher than in the control group (*p* < 0.001).

Table 3 compares the prevalence of kidney stones and family history of kidney stones in women with PCOS and healthy women according to their obesity and T2DM status. There was no statistically significant difference in the prevalence of kidney stones in women with PCOS with BMI < 25 kg/m^2^ in comparison to those PCOS women with BMI ≥ 25 kg/m^2^ (11.1% versus 13.7%; *p* = 0.4). In healthy controls, women with BMI ≥ 25 kg/m^2^ have significantly more history of kidney stones compared to their normal-weight counterpart (9.5% versus 5.8%, *p* = 0.01).

The results of logistic regression models, presented in Table 4, indicate that PCOS status and its phenotypes are associated with an increased risk of kidney stones. In the unadjusted models, women with PCOS had a 70% higher risk of kidney stones compared to healthy controls [OR: 1.70, 95%CI: (1.23,2.33), *p* = 0.001]. Additionally, women with PCOS phenotype A [OR: 2.02, 95%CI: (1.19,3.43), *p* = 0.01] and D [OR: 2.69, 95%CI: (1.16,6.22), *p* = 0.02] had an even higher risk of kidney stones. These findings were consistent even after adjusting for confounding factors.

**Figure 1:**
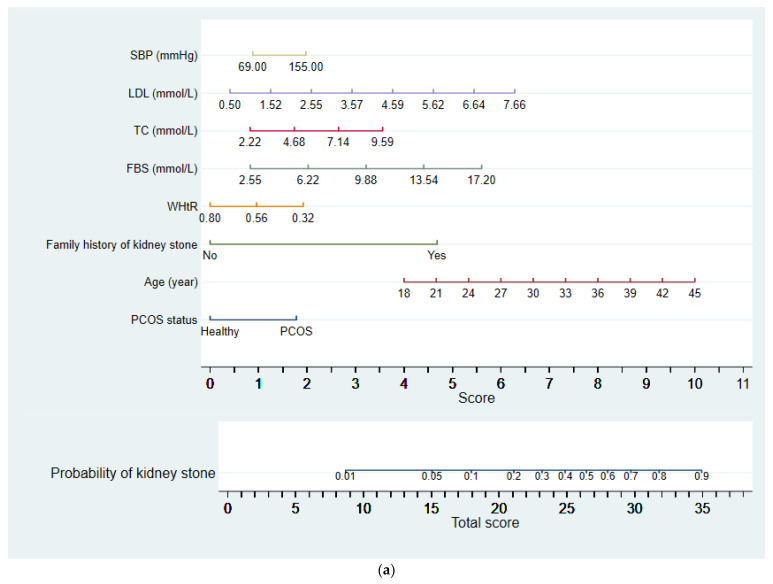

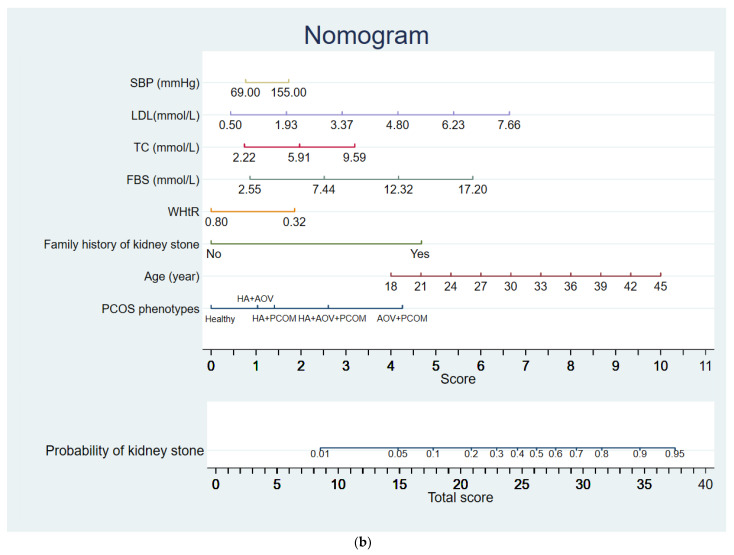
Nomogram prediction models for predicting kidney stones. Nomogram (**a**): polycystic ovarian syndrome (PCOS) status, and nomogram (**b**): PCOS phenotypes.

Figure 1a,b demonstrate the likelihood of kidney stone formation in women with polycystic ovary syndrome (PCOS) by considering the PCOS status and different phenotypes, respectively, while considering seven other risk factors screened out from the aforementioned multivariate logistic regression analysis (SBP, LDL, TC, FBS, WHtR, family history of kidney stone, and age). The total score for each individual is calculated by adding the corresponding scores of each risk factor.

The nomogram converts the total score into a probability of kidney stone occurrence. For instance, a 45-year-old woman with PCOS, a family history of kidney stones, and specific values for other risk factors (waist-to-hip ratio of 0.56, fasting blood sugar of 9.88 mmol/L, total cholesterol of 4.68 mmol/L, low-density lipoprotein of 3.57 mmol/L, and systolic blood pressure of 112 mmHg) would have a total score of 27.7 and a corresponding probability of 57% for kidney stone occurrence.

**Figure 2:**
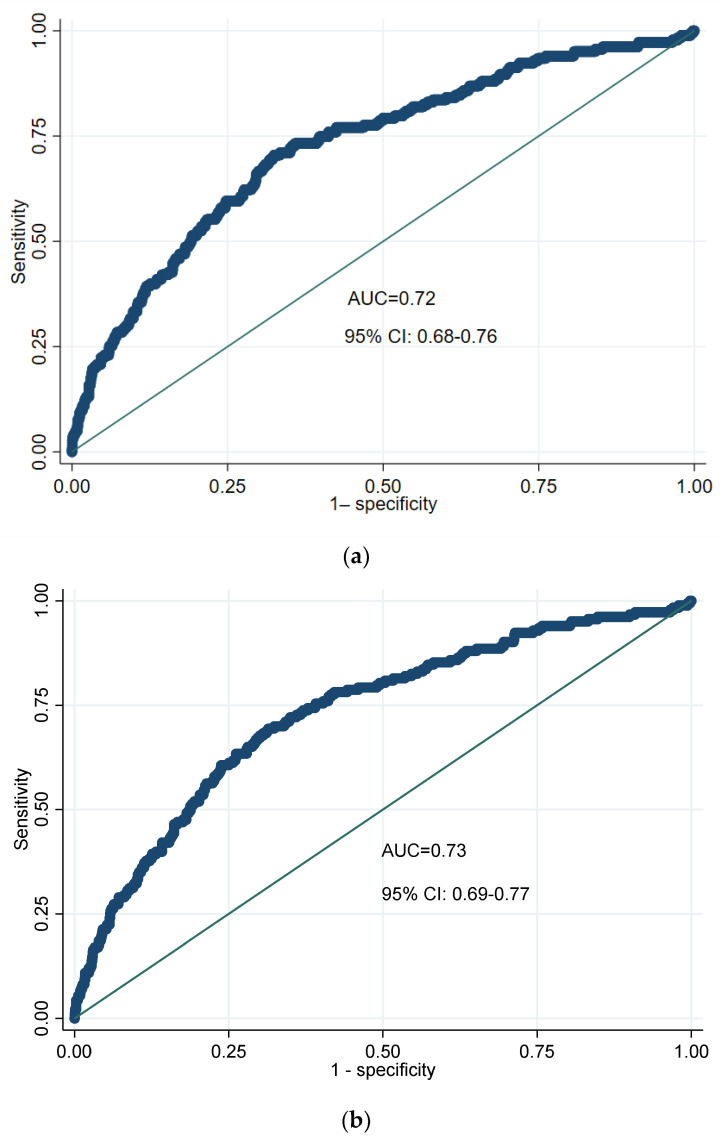
The adjusted ROC curves based on (**a**) PCOS status and (**b**) PCOS phenotypes to predict the risk of kidney stone occurrence.

The AUCs of the model for predicting the risk of kidney stones based on PCOS status and PCOS phenotypes were 0.72 (95% CI: 0.68–0.76) (*p* value = 0.02) and 0.73 (95% CI: 0.69–0.77) (*p* value = 0.03), respectively, as shown in Figure 2a,b. This may indicate that the diagnostic test has discriminatory ability.

**Figure 3:**
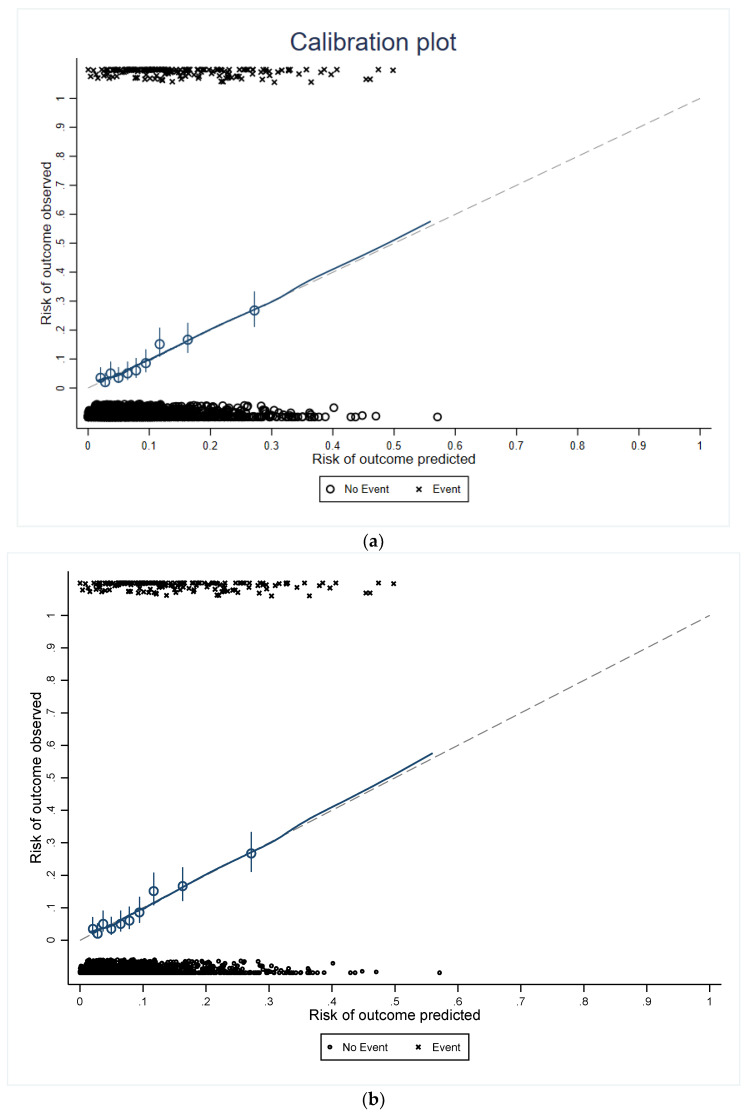
Bootstrap internal validation calibration plot: (**a**) PCOS status and (**b**) PCOS phenotypes. The dashed line indicates the ideal predictions. Points estimated below the dashed line correspond to nomogram over-prediction, while points situated above it correlate with the nomogram under-estimation. Dots indicate the frequency distribution of predicted probabilities.

The external validation of our nomogram was assessed using the Hosmer–Lemeshow test; the goodness of fit with an associated *p* value of 0.08 and 0.1 for two nomogram models allowed for accepting that there are no significant discrepancies between the observed and expected frequencies. The nomogram models were internally verified using the bootstrap test method, where the original data were repeatedly sampled 200 times. The internal validation of our nomograms suggests that the fit of the nomograms is reasonable, as indicated by its very slight departures from a 45-degree slope, which represents the ideal line (Figure 3).

## 4. Discussion

To the best of our knowledge, this study is the first to report the prevalence of kidney stones in women with various phenotypes of Polycystic Ovary Syndrome compared to a healthy control group. Our results indicate that women with PCOS have a 70% increased risk of a history of kidney stones [OR: 1.70, 95%CI: (1.23, 2.33), *p* = 0.001] compared to healthy women. This risk is mainly observed in those women who have all three features of PCOS (OA + HA + PCOM) [OR: 2.02, 95% CI: (1.19–3.43), *p* = 0.01] and those who have menstrual irregularity and polycystic ovarian morphology [OR: 3.03, 95% CI: (1.24–7.41), *p* = 0.01]. Additionally, our model that included PCOS phenotypes had a better predictive power than one only divided into having or not having PCOS.

Nephrolithiasis, commonly known as kidney stones, is a disorder influenced by both intrinsic and environmental factors. Age, sex, and gonadal hormones, such as testosterone and estrogen, play significant roles in the pathogenesis of kidney stones. The prevalence of nephrolithiasis is known to be 2–3 times higher in men of reproductive age compared to women [27]. This has been supported by laboratory findings, such as a study conducted by Nath et al., which found that male stone formers had higher levels of oxalate excretion in their 24 h urinary samples, associated with elevated levels of testosterone [28]. On the other hand, research has shown that estrogen can inhibit the formation of kidney stones by decreasing urinary oxalate excretion [12]. This suggests that postmenopausal women with higher estradiol levels may have a reduced risk of developing calcium oxalate kidney stones [29]. Furthermore, animal studies have also demonstrated a significant decrease in the formation of kidney stones in castrated male rats, providing evidence of the role of gonadal hormones in the development of nephrolithiasis [30]. Despite all this evidence, the relationship between testosterone levels and nephrolithiasis is still in the aura of uncertainty and is a subject of ongoing research. While some studies have reported an association between elevated testosterone levels and an increased risk of kidney stones, other studies have not found any correlation [11,12,24,31,32,33,34].

The prevalence of kidney stones in our study was observed more frequently in phenotypes with menstrual disorders and PCOM features. It is assumed a common underlying mechanism may contribute to the association between PCOS and an increased risk of kidney stones. Menstruation in women is a hormone-dependent process, involving the production and conversion of androgens to estrogen within ovarian follicles. Theca cells in the ovaries produce androgens that are then transported to granulosa cells; in these granulosa cells, androgens are converted to estrogen. Several molecules and proteins are involved in this process, which is under the regulation of the LH hormone [35]. Among the molecules involved in the hormone transportation process, miRNA-125-b plays a key role [36]. MiRNAs are small, non-coding, single-stranded, endogenous molecules that play an important role in the regulation of post-transcriptional gene expression [37]. Interestingly, the role of MicroRNAs in cystic diseases such as PCOS and polycystic kidney disease (PKD) [38,39], as well as kidney stone formation, has been reported in some studies [40].

Recent studies have shown that in women with Polycystic Ovary Syndrome (PCOS), there are pathological changes in the expression of certain MicroRNAs (MiRNAs) in granulosa cells [41,42]. These changes, such as the increased expression of miR-126-5, miR-483, and miR-145, lead to an increased proliferation rate of granulosa cells and increased apoptosis, ultimately leading to higher rates of follicle atresia and a lack of ovulation in women with PCOS [38]. Additionally, studies have also found a potential role for MiRNA expression in the formation of kidney stones, specifically in calcium oxalate kidney stone formation [39,40,41]. These findings have been confirmed in an animal study [43].

In the present study, women who suffer from menstrual irregularities with PCOM (groups A and D) are more at risk of developing kidney stones (approximately two to three times) than healthy women. The findings suggest that the expression disorders of microRNAs may be the underlying cause of both menstrual irregularities or PCOM and kidney stone formation in these women. Since MicroRNA (miRNA) expression is a highly complex phenomenon, this has led to varied findings in studies focused on polycystic ovary syndrome (PCOS) subjects [44]. Notably, serum levels of miRNA-21 and miRNA-6767-5p are elevated, while miRNA-320 is decreased in individuals with PCOS [45,46,47]. These specific miRNAs have been suggested as potential biomarkers for diagnosing PCOS. Additionally, miR-92a and miRNA-93 are relevant in ovarian regulation, displaying critical roles in follicular development and hormonal regulation [48].

The theory suggests that the disruption of microRNAs may serve as a common underlying factor in menstrual disorders accompanied by polycystic ovaries and kidney disorders like kidney stones and polycystic kidneys. The lack of observing a statistically significant correlation between the B and C phenotypes with kidney stones can raise the hypothesis of the absence of a significant role of hyperanrdogenemia in this relationship. Nonetheless, additional investigations are warranted to definitively validate this hypothesis.

The link between PCOS and kidney stones may be partly explained by the potential role of obesity in both diseases [49,50,51]. Obesity may result in nephrolithiasis secondary to insulin resistance and dietary factors [52]; women who weigh more than 220 pounds are 90% more likely to develop kidney stones than those who weigh less than 150 pounds [53]. Additionally, overweight/obese women with PCOS have a greater chance of developing high blood pressure and diabetes, which may lead to kidney stones [54]. Weight loss through dietary interventions and improved insulin resistance has been shown to improve various features of PCOS, possibly kidney stones [55]. In the present study, the adverse effect of obesity on kidney stones was observed in healthy controls, but not in women with PCOS (Table 3).

Our study has several strengths, including the utilization of a population-based sample, as opposed to recruitment solely from a tertiary center. Additionally, all women, regardless of their menstrual patterns or features of androgen excess, were evaluated for biochemical hyperandrogenism and polycystic ovarian morphology using national cut-offs for androgen hormones in the definition of hyperandrogenism.

There are also several limitations to the study. The main limitation of the present study is the lack of a comprehensive assessment of kidney stones. The data used in this study were primarily obtained through self-reported patient information; the type and composition of kidney stones, 24 h urinary samples, and the function of the parathyroid gland were not evaluated, since different types of stones have different pathogenetic mechanisms. Additionally, this study design may be subjected to recall bias in terms of the prevalence of nephrolithiasis and family history of kidney stones; moreover, PCOS women and healthy control women were not comparable for this family history even through adjustment in multivariable analysis. The other limitation is the absence of an ultrasonographic evaluation of the urinary tract system for virgin cases, as sociocultural constraints prevented us from conducting transvaginal ultrasonography to determine the presence of polycystic ovaries. Testosterone was measured using the enzyme immunoassay method instead of highly efficient liquid chromatography–tandem mass spectrometry (LC-MS/MS), which may influence the accuracy of our assessment. Furthermore, our results were not adjusted for dietary behavior. We do not have an adequate number of diabetes among our study groups to investigate the interaction effect of diabetes on kidney stones. Our control group was younger than PCOS ones; therefore, we are unable to precisely interpret the interaction effect of cardio-metabolic complications that are usually more observed in women with PCOS.

## 5. Conclusions

The study findings indicate that women with Polycystic Ovary Syndrome (PCOS) have an increased risk of developing kidney stones, particularly those with menstrual irregularities accompanied by PCOM. These findings need to be interpreted with caution due to the lack of comprehensive assessment of kidney stones in the present study. The findings of the present study are too premature to deduce a solid conclusion; however, it may raise awareness of healthcare providers in terms of considering the possibility of increasing the occurrence of kidney stones in women with PCOS. To further understand the underlying mechanisms, comprehensive longitudinal studies are highly recommended to further investigate this association in various ethnicities with different PCOS features and the possible pathways linking PCOS and kidney stones.

## Figures and Tables

**Table 1:** Characteristics of women with PCOS and healthy women.

	PCOS (*n* = 520)	Healthy Women (*n* = 1638)	*p*-Value
Age, year, mean, SD	26.0 (7.3)	28.4 (8.2)	<0.001
BMI (kg/m^2^)	25.7 (5.0)	25.7 (4.8)	0.9
History kidney stone (yes/no)	65 (12.5)	127 (7.7)	0.001
Family history of kidney stone (yes/no)	257 (49.4)	520 (31.7)	<0.001
Education (diploma and upper)	216 (41.8)	818 (50.0)	0.001
Physical activity (>600)	152 (32.9)	464 (30.0)	0.2
HC, cm	100.1 (9.4)	100.6 (9.2)	0.3
WC, cm	80.0 (11.7)	81.0 (11.5)	0.09
WHR	0.80 (0.07)	0.80 (0.07)	0.1
WHtR	0.50 (0.08)	0.51 (0.07)	0.02
ABSI	0.73 (0.70–0.77)	0.74 (0.71–0.78)	0.001
VAI	1.63 (1.12–2.59)	1.63 (1.08–2.76)	0.8
LAP	21.5 (11.2–41.9)	22.9 (12.2–43.8)	0.2
#Hormones, mean (SD) or median (25–75%)			
LH, mIU/mL	5.85 (3.6–9.1)	4.6 (3.3–6.4)	<0.001
FSH, mIU/mL	7.0 (5.2–9.1)	7.6 (5.75–10.1)	<0.001
OH17 OH-P, nmol/L	1.7 (1.0–2.5)	1.3 (0.9–1.9)	0.01
TT, ng/dL	0.57 (0.30–0.83)	0.32 (0.14–0.57)	<0.001
FAI	1.18 (0.60–1.99)	0.51 (0.24–0.96)	<0.001
A4, ng/mL	1.8 (1.0–2.3)	1.1 (0.9–1.7)	<0.001
DHEAS, mg/DI	159.6 (93.7–213.3)	119.3 (63.7–176.6)	<0.001
SHBG, nmol/L	45.6 (34.0–62.4)	59.7 (44.3–84.9)	<0.001
#Cardio-metabolic factors, mean (SD) or median (25–75%)			
FBG (mmol/L)	4.8 (4.5–5.0)	4.8 (4.5–5.0)	0.8
Bs-2hPG (mmol/L)	5.4 (4.7–6.3)	5.5 (4.7–6.4)	0.6
TC (mmol/L)	4.45 (3.88–5.20)	4.60 (3.98–5.30)	0.04
TG (mmol/L)	1.07 (0.81–1.50)	1.06 (0.79–1.59)	0.9
HDL-C (mmol/L)	1.19 (1.0–1.37)	1.18 (1.0–1.37)	0.9
LDL-C (mmol/L)	2.72 (2.21–3.34)	2.86 (2.33–3.44)	0.01
SBP (mmHg)	105.5 (11.8)	107.0 (11.5)	0.01
DBP (mmHg)	71.3 (9.4)	72.2 (9.2)	0.06

Values are presented as mean (SD), median (25–75%), or number (percentage) as appropriate. PCOS, Polycystic ovary syndrome; BMI, Body mass index; HC, hip circumference; WC, Waist circumference; WHR, waist-to-hip ratio; WHtR, waist-to-height ratio; ABSI, a body shape index; VAI, visceral adiposity index; LAP, lipid accumulation product. LH, luteinizing hormone; FSH, follicle-stimulating hormone; 17OH-P, 17-hydroxyprogesterone; TT, Total testosterone; FAI, free androgen index; A4, androstenedione; DHEAS, dehydroepiandrosterone sulfate; SHBG, sex hormone binding globulin; FBG, fasting blood glucose; Bs-2hPG, 2 h plasma glucose; TC, total cholesterol; TG, triglycerides; HDL, high-density lipoprotein; LDL, low-density lipoprotein; SBP, systolic blood pressure; DBP, diastolic blood pressure.

**Table 2:** Demographic characteristics of women with different PCOS phenotypes and healthy women.

	PCOS Phenotype A (OA + HA + PCOM) (*n* = 124)	PCOS Phenotype B (OA + HA) (*n* = 250)	PCOS Phenotype C (HA + PCOM) (*n* = 108)	PCOS Phenotype D (OA + PCOM) (*n* = 38)	Healthy Women (*n* = 1638)	*p*-Value
Age, year, mean, SD	25.5 (6.5) H	26.5 (7.6) H	25.3 (7.1) H	26.7 (7.9)	28.4 (8.2)	<0.001
BMI (kg/m^2^)	25.8 (4.5)	26.2 (5.5)	25.1 (4.7)	24.5 (4.0)	25.7 (4.8)	0.2
History kidney stone (yes/no)	18 (14.5) H	26 (10.4)	14 (13.0) H	7 (18.5) H	127 (7.7)	0.01
Family history of kidney stone (yes/no)	59 (47.5) H	126 (50.4) H	54 (50.0) H	18 (47.4) H	520 (31.7)	<0.001
Education (diploma and upper)	60 (48.4)	91 (36.5) H	48 (45.3)	17 (44.5)	818 (50.0)	0.01
Physical activity (>600 min/week)	29 (26.6)	85 (37.3)	25 (27.5)	13 (38.2)	464 (30.0)	0.1
HC, cm	99.9 (8.9)	101.0 (10)	98.9 (8.9)	98.5 (8.3)	100.6 (9.2)	0.2
WC, cm	80.3 (11.5)	80.8 (11.9)	78.7 (11.6)	77.7 (11.0)	81.0 (11.5)	0.1
WHR	0.80 (0.07)	0.80 (0.08)	0.79 (0.07)	0.79 (0.07)	0.80 (0.07)	0.4
WHtR	0.51 (0.07)	0.51 (0.08)	0.49 (0.08)	0.49 (0.07)	0.51 (0.07)	0.08
ABSI	0.73 (0.70–0.77) H	0.73 (0.70–0.77) H	0.73 (0.69–0.76) H	0.73 (0.70–0.77)	0.74( 0.71–0.78)	0.01
VAI	1.7 (1.1–2.7)	1.7 (1.11–2.6)	1.45 (1.12–2.23)	1.68 (1.12–3.28)	1.63 (1.1–2.8)	0.5
LAP	23.2 (14.7–42.2)	21.6 (11.7–43.9)	18.6 (9.9–29.8)	21.0 (8.3–45.0)	22.9 (12.2–43.8)	0.1
#Hormones, mean (SD) or median (25–75%)						
LH, mIU/mL	5.3 (3.6–8.6) H	5.9 (3.5–9) H	6.8 (4.2–9.9) HDBA	5.1 (3.3–7.5)	4.6 (3.3–6.4)	<0.001
FSH, mIU/mlL	6.9 (4.5–9.1) H	7.0 (5.2–9.1) H	7.2 (5.0–9.3) H	7.0 (5.1–9.2) H	7.6 (5.7–10.1)	0.001
OH17 OH-P, nmol/L	1.3 (0.8–2.1)	1.8 (1.2–3.0)HA	1.7 (1.1–2.3)	1.6 (1.1–3.1)	1.3 (0.9–1.9)	0.01
TT, ng/dL	0.4 (0.3–0.8) H	0.6 (0.3–0.8) H	0.5 (0.3–0.8) H	0.5 (0.3–0.8) H	0.3 (0.1–0.6)	0.001
FAI	1.1 (0.6–1.8) H	1.4 (0.6–2.1) HDC	1.0 (0.5–1.7) H	0.9 (0.6–1.3) H	0.5 (0.2–1.0)	0.001
A4, ng/mL	1.6 (0.9–2.3) H	1.9 (1.0–2.4) H	1.8 (1.1–2.3) H	1.8 (1.1–2.4) H	1.1 (0.9–1.7)	0.001
DHEAS, mg/DI	146.9 (80–216.6) H	171.8 (101.2–214) HCA	150 (86.8–209.1) H	167.9 (117.7–224.6) H	119.3 (63.7–176.6)	0.001
SHBG, nmol/L	44.1 (33.7–60.3) H	45.0 (33.0–58.6) H	47.5 (34.9–60.2) HDAB	61.9 (46.8–78.0)	59.7 (44.3–84.9)	0.001
#Cardio-metabolic factors, mean (SD) or median (25–75%)						
FBG (mmol/L)	4.8 (4.5–5.0)	4.8 (4.5–5.0)	4.7 (4.5–5.0)	4.7 (4.4–5.0)	4.8 (4.5–5.0)	0.3
Bs-2 h PG (mmol/L)	5.3 (4.6–6.2)	5.6 (4.8–6.5)	5.3 (4.6–5.9)	5.4 (4.8–6.2)	5.5 (4.7–6.4)	0.4
TC (mmol/L)	4.5 (3.8–5.2)	4.5 (4.0–5.2)	4.3 (3.8–5.0) HB	4.2 (3.6–4.9) HB	4.6 (4.0–5.3)	0.05
TG (mmol/L)	1.1 (0.8–1.7)	1.1 (0.8–1.5)	1.0 (0.8–1.3)	1.1 (0.7–1.7)	1.1 (0.8–1.6)	0.2
HDL-C (mmol/L)	1.2 (1.0–1.4)	1.2 (1.0–1.3)	1.2 (1.0–1.4)	1.0 (0.9–1.3)	1.2 (1.0–1.4)	0.4
LDL-C (mmol/L)	2.6 (2.1–3.3) H	2.8 (2.3–3.4)	2.6 (2.2–3.3) H	2.5 (2.1–3.2) H	2.9 (2.3–3.4)	0.04
SBP (mmHg)	105.9 (11.1)	105.5 (12.4)	104.6 (11.5)	106.6 (11.1)	107.1 (11.5)	0.09
DBP (mmHg)	70.9 (9.0)	71.7 (10.0)	71.0 (8.6)	70.6 (8.7)	72.2 (9.2)	0.3

Values are presented as mean (SD), median (25–75%), or number (percentage) as appropriate. Bonferroni post hoc test or Dunn test was used for significant ANOVA and Kruskal–Wallis variables, as appropriate. A: Phenotype A; B: Phenotype B; C: Phenotype C; D: Phenotype D; and H: Healthy women. PCOS, Polycystic ovary syndrome; OA, oligo anovulation; HA, hyperandrogenism; PCOM, polycystic ovaries morphology; BMI, Body mass index; HC, hip circumference; WC, Waist circumference; WHR, waist-to-hip ratio; WHtR, waist-to-height ratio; ABSI, a body shape index; VAI, visceral adiposity index; LAP, lipid accumulation product. LH, luteinizing hormone; FSH, follicle-stimulating hormone; 17OH-P, 17-hydroxyprogesterone; TT, Total testosterone; FAI, free androgen index; A4, androstenedione; DHEAS, dehydroepiandrosterone sulfate; SHBG, sex hormone binding globulin; FBG, fasting blood glucose; Bs-2hPG, 2 h plasma glucose; TC, total cholesterol; TG, triglycerides; HDL, high-density lipoprotein; LDL, low-density lipoprotein; SBP, systolic blood pressure; DBP, diastolic blood pressure. PCOS phenotypes: Phenotype A: those who had all three features of PCOS (OA + HA + PCOM), phenotype B: those who had two OA and HA features of PCOS (OA + HA), phenotype C: those who had two features of PCOS HA + PCOM, phenotype D: those who had OA+ PCOM features of PCOS.

**Table 3:** Prevalence of kidney stones and family history of kidney stones in women with PCOS and healthy women according to their obesity and T2DM status.

	PCOS (*n* = 520)		*p*-Value	Control (*n* = 1638)		*p*-Value	P_a_	P_b_
	BMI < 25 kg/m^2^ (*n* = 243)	BMI ≥ 25 kg/m^2^ (*n* = 277)		BMI < 25 kg/m^2^ (*n* = 760)	BMI ≥ 25 kg/m^2^ (*n* = 878)			
Kidney stones	27 (11.1%)	38 (13.7%)	0.4	44 (5.8%)	83 (9.5%)	0.01	0.005	0.04
Family history of kidney stones	124 (51%)	133 (48%)	0.5	238 (31.3%)	282 (32.1%)	0.7	<0.001	<0.001
	PCOS (*n* = 520)		*p*-value	Control (*n* = 1638)		*p*-value	P_c_	P_d_
	T2DM^+^ (*n* = 55)	T2DM^−^ (*n* = 465)		T2DM^+^ (*n* = 134)	T2DM^−^ (*n* = 1504)			
Kidney stones	9 (16.4%)	56 (12%)	0.4	12 (9%)	115 (7.7%)	0.6	0.1	0.003
Family history of kidney stones	29 (52.7%)	228 (49%)	0.6	42 (31.3%)	478 (31.8%)	0.9	0.01	<0.001

PCOS, Polycystic ovary syndrome; T2DM, type 2 diabetes mellitus; P_a_, PCOS < 25 kg/m^2^ versus control <25; P_b_, PCOS ≥ 25 kg/m^2^ versus control ≥25; P_c_, PCOS with T2DM^+^ versus control T2DM^+^; P_d_, PCOS without T2DM versus control without T2DM.

**Table 4:** Unadjusted/adjusted logistic regression models for the effect of PCOS status and its phenotypes on the history of kidney stone outcome.

	Model 1			Model 2		
	OR	95% CI	*p*-Value	OR	95% CI	*p*-Value
PCOS	1.70	1.23–2.33	0.001	1.59	1.12–2.25	0.01
PCOS phenotype						
PCOS Phenotype A (OA + HA + PCOM)	2.02	1.19–3.43	0.01	1.97	1.09–3.55	0.02
PCOS Phenotype B (OA + HA)	1.38	0.88–2.15	0.1	1.31	0.82–2.10	0.3
PCOS Phenotype C (HA + PCOM)	1.77	0.98–3.20	0.06	1.44	0.75–2.80	0.3
PCOS Phenotype D (OA + PCOM)	2.69	1.16–6.22	0.02	3.03	1.24–7.41	0.01

Reference is healthy group. Model 1: unadjusted; Model 2: adjusted for age, family history of kidney stones, PCOS, Polycystic ovary syndrome; OA, oligo anovulation; HA, hyperandrogenism; PCOM, polycystic ovaries morphology. PCOS phenotypes: Phenotype A: those who had all three features of PCOS (OA + HA + PCOM), phenotype B: those who had two OA and HA features of PCOS (OA + HA), phenotype C: those who had two features of PCOS HA + PCOM, phenotype D: those who had OA+ PCOM features of PCOS.

## Data Availability

The data presented in this study are available on request from the corresponding author.
